# Detection of maize stem diameter by using RGB-D cameras’ depth information under selected field condition

**DOI:** 10.3389/fpls.2024.1371252

**Published:** 2024-04-22

**Authors:** Jing Zhou, Mingren Cui, Yushan Wu, Yudi Gao, Yijia Tang, Bowen Jiang, Min Wu, Jian Zhang, Lixin Hou

**Affiliations:** ^1^ College of Information Technology, Jilin Agricultural University, Changchun, China; ^2^ Faculty of Agronomy, Jilin Agricultural University, Changchun, China; ^3^ Department of Biology, University of British Columbia, Okanagan, Kelowna, BC, Canada

**Keywords:** crop phenotyping, RGB-D, depth information, field maize, stem diameter

## Abstract

Stem diameter is a critical phenotypic parameter for maize, integral to yield prediction and lodging resistance assessment. Traditionally, the quantification of this parameter through manual measurement has been the norm, notwithstanding its tedious and laborious nature. To address these challenges, this study introduces a non-invasive field-based system utilizing depth information from RGB-D cameras to measure maize stem diameter. This technology offers a practical solution for conducting rapid and non-destructive phenotyping. Firstly, RGB images, depth images, and 3D point clouds of maize stems were captured using an RGB-D camera, and precise alignment between the RGB and depth images was achieved. Subsequently, the contours of maize stems were delineated using 2D image processing techniques, followed by the extraction of the stem’s skeletal structure employing a thinning-based skeletonization algorithm. Furthermore, within the areas of interest on the maize stems, horizontal lines were constructed using points on the skeletal structure, resulting in 2D pixel coordinates at the intersections of these horizontal lines with the maize stem contours. Subsequently, a back-projection transformation from 2D pixel coordinates to 3D world coordinates was achieved by combining the depth data with the camera’s intrinsic parameters. The 3D world coordinates were then precisely mapped onto the 3D point cloud using rigid transformation techniques. Finally, the maize stem diameter was sensed and determined by calculating the Euclidean distance between pairs of 3D world coordinate points. The method demonstrated a Mean Absolute Percentage Error (*MAPE*) of 3.01%, a Mean Absolute Error (*MAE*) of 0.75 mm, a Root Mean Square Error (*RMSE*) of 1.07 mm, and a coefficient of determination (*R*²) of 0.96, ensuring accurate measurement of maize stem diameter. This research not only provides a new method of precise and efficient crop phenotypic analysis but also offers theoretical knowledge for the advancement of precision agriculture.

## 1 Introduction

The global population has now surpassed 8 billion and is projected to reach more than 9 billion by the year 2050 (Rahimifard et al., 2013). This necessitates an increase in crop yield by 70% in order to meet the growing global food requirements (Wang, 2022). However, agricultural production is facing unprecedented challenges including global climate change, natural disasters, and intense human activities, making the acceleration of breeding research particularly crucial. In recent years, as the cost of gene sequencing has steadily decreased and its speed has increased, agronomic experts have collected a vast array of crop genotypic information. Nevertheless, over the past few decades, the development of crop phenotyping technologies has lagged behind (Shen et al., 2022). In particular, the capacity for precise measurement of small-sized phenotypes in open field environments is relatively limited, requiring a substantial amount of manual labor. This method is not only costly but also inefficient. Particularly under conditions of high temperatures, intense light, and long work periods, the subjectivity and potential for error in data measurement can increase significantly. Therefore, it is essential to research crop phenotyping monitoring technologies that offer a relatively higher degree of automation and measurement accuracy with lower costs.

Maize (*Zea mays* L.) is one of the most important cereal crops in the world, distinguished by its prodigious productivity, substantial nutritive value, and amenability to biotechnological interventions. Such characteristics render it a model crop for diverse applications, ranging from alimentation to scientific investigation and bioenergy production (Bothast and Schlicher, 2005; Duvick, 2005; Nuss and Tanumihardjo, 2010). In the array of phenotypic characteristics of maize, stem diameter assumes a pivotal role, serving not only as an indicator for forecasting yield and assessing lodging resistance but also as a predictive measure for the seasonal biomass accumulation in maize (Kelly et al., 2015; Mousavi et al., 2020; Liu et al., 2022). Employing non-invasive imaging techniques for the *in situ* measurement of maize stem diameter could substantially improve the efficiency of breeding research. Batz et al. (2016) utilized a dual-camera system composed of red-green-blue (RGB) and time-of-flight (TOF) cameras to capture images of indoor-grown sorghum plants. The actual stem diameter was deduced from these images by applying a pixel length conversion factor, yielding an *R*² of 0.70. Notwithstanding, incongruities in the field of view (FOV) between RGB and TOF cameras can result in disparate positioning of the same object within each camera’s perspective. This discrepancy poses challenges for the accurate alignment of RGB and TOF images, a problem that remains unresolved. Zhang and Grift (2012) utilized a sensor comprising a charge-coupled device (CCD) camera in conjunction with an oblique laser sheet to image Miscanthus stems. They accurately measured stem diameters using 2D image processing methods grounded in the principles of pinhole imaging, achieving an *R*² of 0.926. Despite the widespread application of 2D image processing technology in crop phenotyping, it presents constraints when characterizing phenotypic parameters in 3D space. Therefore, the fusion of 2D image processing with depth-perception technologies is expected to enhance the accuracy and reliability in the acquisition of crop phenotypic parameters (Chene et al., 2012; Wang and Li, 2014; Malik et al., 2019). Xu et al. (2023) captured color, depth, and near-infrared (NIR) images of cucumber seedlings within a controlled greenhouse setting employing dual Azure Kinect depth cameras. Segmentation of the foliage and stem components was accomplished through the application of a Mask R-CNN framework on the NIR images. Leveraging the approximate rectangular characteristic of cucumber seedling stems and incorporating depth information, researchers have computed the stem diameter of these seedlings. The *R*² exceeded 0.82. The experimental environment of this study is controllable, with the effects of ambient light, shadows, and wind being negligible, providing ideal conditions for crop phenotyping monitoring. Additionally, in controlled environment potted crop phenotyping systems, not only can environmental factors be precisely regulated, but efficient and accurate phenotypic analyses are often performed through the application of an electric turntable or a scanning device integrated with a stepper motor, further enhancing the precision of data collection (Wang and Chen, 2020; Arief et al., 2021).

Although the indoor experimental setting offers precise control over variables such as light, temperature, and background, thus creating nearly ideal conditions for the accurate measurement and analysis of crop phenotypes, the complexity and unpredictability of outdoor environments pose challenges to crop phenotyping analysis. Nevertheless, the analysis of crop phenotypes under field environments is confounded by a multiplicity of variables, including fluctuations in lighting conditions, topographical variation, and variations in plant density. During the initial phases of crop growth, top-view RGB imaging is employed to analyze the phenotypic characteristics of crops in open field environments (Liu et al., 2017; Zhou et al., 2018; Qiu et al., 2021). Li et al. (2021) acquired top-view images of maize seedlings with an EOS5DIII digital camera and employed convolutional neural network (CNN) algorithms to separate the seedlings from their background. Morphological features of the maize seedlings were then extracted using edge detection, connective domain markers, and morphological operations. Furthermore, this research transformed the RGB data of the images into the hue saturation value (HSV) color model to facilitate the extraction of the colorimetric properties of the seedlings. Concurrent with the rapid growth of crops during their initial stages, side-view imaging technology is being progressively utilized for phenotypic analysis in open field environments (Baharav et al., 2017; Song et al., 2019). Qiao et al. (2022) acquired side-view images of red jujube tree trunks utilizing an Intel RealSense D435i camera and separated the trunks from the background using an improved neural network model and the Maximum Between-Class Variance (Otsu) algorithm. The pixel stem diameter of the red jujube tree trunks was measured using the Euclidean distance, and the actual stem diameter was calculated based on depth information and intrinsic camera parameters, with an average absolute error of 5.02 mm. The study capitalized on the prominent linear features of red jujube trunks to extract skeletal information from crop images for stem diameter estimation, resulting in a high level of precision. Furthermore, the experiment necessitated considerable computational capacities, entailing elevated operational processing demands.

In previous studies, we primarily relied on the use of external reference objects, such as chessboards, in combination with RGB images from RGB-D cameras for measuring the diameter of maize stems. Although this method is simple and effective, it is complex to operate in the field and susceptible to external environmental influences (Zhou et al., 2023a, Zhou et al., 2023b). This study proposes a novel measurement method that aligns RGB and depth images and utilizes back-projection technology to convert 2D coordinates into 3D spatial coordinates. This makes it possible to precisely measure the diameter of maize stems without the need for external reference objects. Furthermore, we directly extracted the necessary key information from 2D images and mapped the 3D coordinates into the 3D point cloud, avoiding complex processing of large volumes of 3D point cloud data. While maintaining measurement accuracy, this approach reduces the computational burden. This method not only simplifies the field measurement process but also reduces the reliance on high-cost equipment and complex data processing, significantly lowering the economic cost of research. Furthermore, it offers an efficient and accurate pathway for digital agriculture and crop phenotypic analysis.

## 2 Materials and methods

### 2.1 System architecture

**Figure 1:**
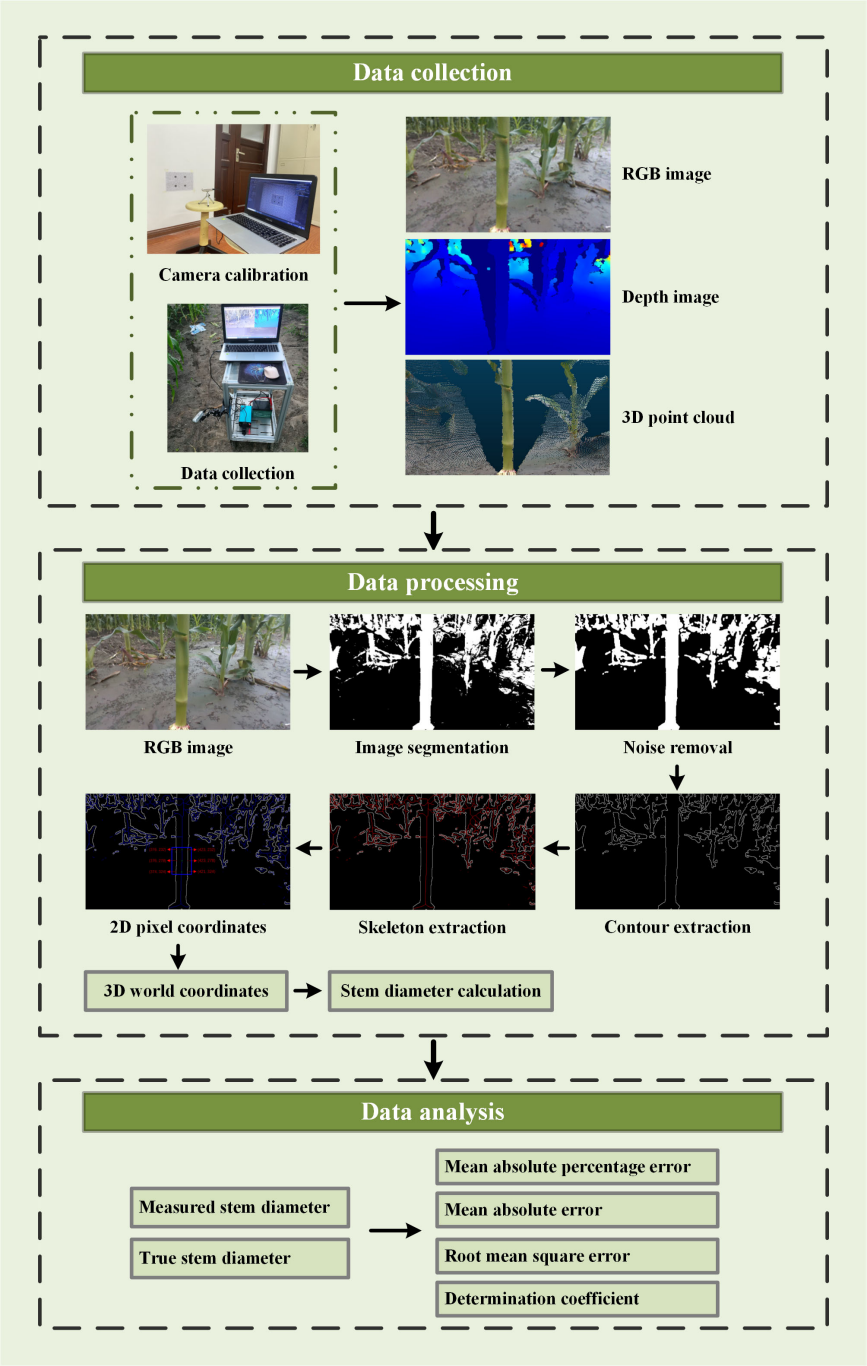
Scheme of system architecture for acquisition of field maize stem diameter using depth information.

The acquisition of field maize stem diameter using depth information from an RGB-D camera can be divided into three parts: data collection, data processing, and data analysis. The architecture of the system is shown in **Figure 1**.

### 2.2 Camera calibration

**Figure 2:**
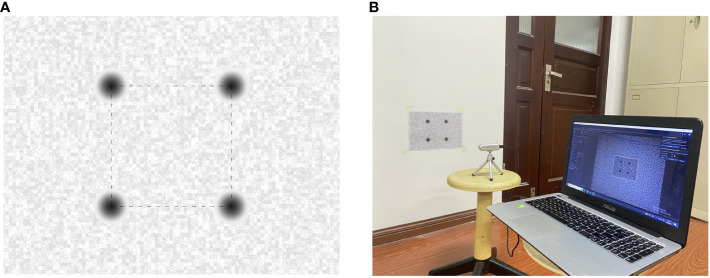
Standard calibration target and scene **(A)** Standard calibration Target **(B)** Camera calibration scene.

**Figure 3:**
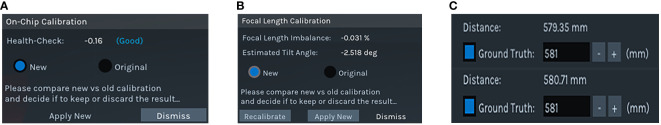
Calibration results **(A)** On-chip calibration result **(B)** Focal length calibration result **(C)** Comparison before and after tare calibration.

Despite the Intel RealSense D435i camera is precisely self-calibrated before shipment, the operation in a cornfield environment, which involves prolonged exposure to high temperatures and intense sunlight, may compromise its accuracy. Accordingly, it is imperative to undertake self-calibration of the camera. The Depth Quality Tool v2.54.1 was employed for on-chip calibration, focal length calibration, and tare calibration. On-chip calibration is primarily aimed at reducing noise in depth data, and focal length calibration is performed to correct distortions in depth maps that result from focal length imbalances. Tare correction is implemented to enhance the precision of depth measurements. After completing the on-chip calibration and focal length calibration, two key metrics can be observed: health-check and focal length imbalance. If the health-check value is below 0.25 and the focal length imbalance is within ±0.2%, it can be concluded that the camera calibration data are normal, and no update is required (Grunnet-Jepsen et al., 2021). In the course of the calibration process, a standard calibration target of A4 dimensions is employed. This target features a dashed square with side lengths of 0.1 meters. Illustrations of the standard calibration target and the camera calibration scene are depicted in **Figure 2**. The results of the on-chip calibration, focal length calibration, and tare calibration are presented in **Figure 3**.

**Figures 3A**, **B** demonstrate that the obtained health-check value and the focal length imbalance value are -0.16 and -0.031%, respectively. These values fall within the established normal range, thus obviating the necessity for updates to the on-chip calibration and focal length calibration data. In addition, **Figure 3C** illustrates the efficacy of tare calibration, whereby the measurement error was reduced from 1.65 millimeters pre-calibration to 0.29 millimeters post-calibration. Despite the depth error reduction being modest at 1.36 millimeters, the absolute discrepancy between the actual and measured diameters of maize stems—critical in the context of measuring tasks—is at the millimeter scale. Consequently, the implementation of this calibration process is essential.

### 2.3 Data collection

Field trials were carried out at the teaching and research base of Jilin Agricultural University in Changchun, Jilin Province, China. The experimental subjects were maize plants at the small bell stage, with the maize variety being Ji Dan 27. Inter-plant spacing was maintained at 0.4 meters, and inter-row spacing at 0.8 meters. This planting pattern is designed to enhance the convenience of experimental operations while minimizing physical interference between plants by optimizing spatial distribution. The experimental plot spanned an area of 160 meters by 100 meters, corresponding to a planting density of 50,000 plants per hectare. Image acquisition commenced on the 50th day after sowing. The collection activity was scheduled between 15:00 and 18:00 in July 2023, under clear weather with occasional cloudiness. Imagery was acquired from six randomly chosen rows of maize within the experimental plot. In the early stages of crop growth, overlapping of plant canopies was minimal, allowing for the assumption that canopy density exerted an insignificant influence on the data collection.

In the experimental setup, data acquisition was facilitated by an array of instruments, comprising an Intel RealSense D435i camera, a vehicle-mounted mobile platform, a battery with a capacity of 12 ampere-hours (AH), an electrical power inverter, and a laptop computer. The camera was mounted on the vertical frame of a self-designed vehicle platform using a tri-axial adjustment arm. To reduce the influence of adjacent plants and weeds on data collection, the camera was positioned at a 45-degree downward angle to capture images of the maize stems. The camera is operational within a proximal range of 0.3 meters to 3 meters, ensuring optimal function. To ensure full morphological documentation of maize stems, the apparatus is positioned at a distance of 0.6 meters from the base of the maize plants with an elevation of 0.5 meters above the ground level. The energy supply for field operations is provided by a 12AH battery, which, through an inverter, furnishes a consistent power source to a laptop computer. This configuration is designed to guarantee uninterrupted laptop functionality in diverse field conditions.

**Figure 4:**
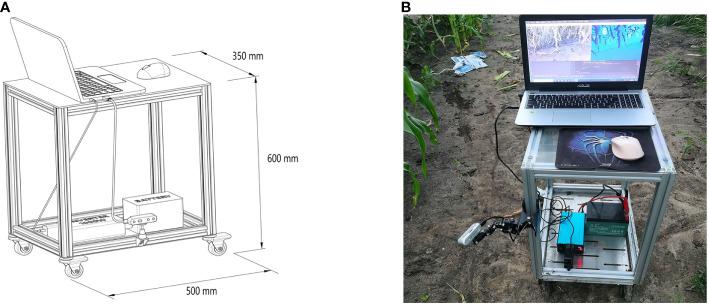
Field-based mobile measuring platform: **(A)** Schematic of the mobile measuring platform **(B)** Photograph of the actual mobile measuring platform.

**Figure 5:**
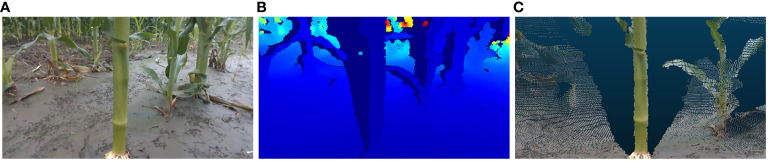
Maize stem information acquisition: **(A)** RGB image **(B)** Depth map **(C)** 3D point cloud.

The laptop in question is configured with the Windows 10 operating system and is equipped with Python 3.10 programming environment and Intel RealSense Viewer v2.54.1. In the Python environment, the camera simultaneously acquired RGB and depth images at fixed poses and generated 3D point clouds of maize stems using the Intel RealSense Viewer. These point clouds were then loaded into the CloudCompare software for visualization. The resolution of 848×480 was selected for acquiring both RGB and depth images, as this resolution has been demonstrated to yield the highest quality of depth information from the camera (Grunnet-Jepsen et al., 2018). A schematic representation of the data acquisition apparatus is depicted in **Figure 4**. Illustrative examples of the acquired RGB images, depth maps, and 3D point clouds are shown in **Figure 5**.

### 2.4 Data processing

#### 2.4.1 Image alignment

**Figure 6:**
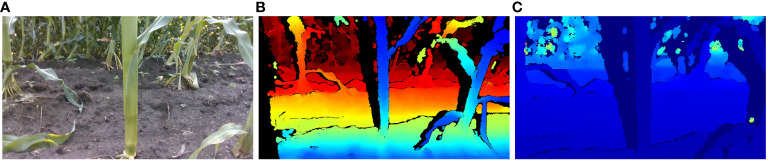
Comparative images before and after alignment: **(A)** RGB image of maize stem **(B)** Depth image before alignment **(C)** Depth image after alignment.

Techniques for image alignment are principally bifurcated into two categories: the adjustment of RGB images for conformity with depth images, and conversely, the rectification of depth images to align with RGB counterparts. Given the broader field of view of the depth camera compared to the RGB camera on the Intel RealSense D435i, aligning RGB images to depth images can result in data loss or the occurrence of voids in the aligned RGB images. To obviate these impediments, this study employs a method that leaves the RGB images unaltered while aligning the depth images to them, thereby accomplishing the image alignment process. In the Python programming environment, the alignment of images was executed by employing the rs.align class within the pyrealsense2 library. This method produced alignment between depth and color frames, applicable for both RGB and depth image analysis. A comparative illustration of the images before and after alignment is presented in **Figure 6**.

#### 2.4.2 Image preprocessing

In this study, the combined HSV and Otsu algorithm was employed to discriminate the principal maize stems from complex field backgrounds. The OpenCV library’s cv.cvtColor and cv.threshold functions were utilized for this task, with the OpenCV library operating on version 4.8.0. Furthermore, the morphological internal gradient algorithm was employed to obtain the contours of maize stems, facilitated by the functions cv.morphologyEx and cv.subtract. Given the established validation of the aforementioned algorithms in antecedent studies, detailed exposition is eschewed in this research (Zhou et al., 2023a, Zhou et al., 2023b).

#### 2.4.3 Skeleton extraction algorithm

In the realm of image processing technology, the task of delineating and distilling salient features from intricate image compositions holds paramount significance. Skeletonization is employed as a strategy for the abstraction of morphological characteristics, and is widely acknowledged as an efficacious approach for feature delineation. Presently, methods that incorporate skeletonization algorithms to discern target features have found widespread application across various sectors, including industrial inspection, medical diagnostics, and crop phenotypic analysis (Patel et al., 2012; Jin and Saha, 2013; Liu et al., 2021). In the domain of crop phenotyping, extracting the skeletal structure of crop stems and utilizing this skeleton to assist in the measurement of stem diameter simplifies the complexity involved in such measurements. Furthermore, this approach enhances the automation of measuring stem diameter (Qiao et al., 2022).

Skeleton extraction algorithms can be primarily categorized into three predominant groups: those that utilize distance transformation, those employing thinning algorithms, and those founded on Voronoi diagrams (Jin and Kim, 2017). Skeleton extraction algorithms based on distance transformation can generate smoother and more continuous skeletons but may overlook certain details. Methods based on Voronoi diagrams may extract numerous false skeleton branches and are computationally intensive. Relative to alternative approaches, skeleton extraction algorithms that employ thinning techniques are proficient in generating refined skeletons for elongate structures (Chen et al., 2011). Thus, for the analysis of elongated maize stems, an algorithm based on thinning for skeletonization may be a superior choice.

**Figure 7:**
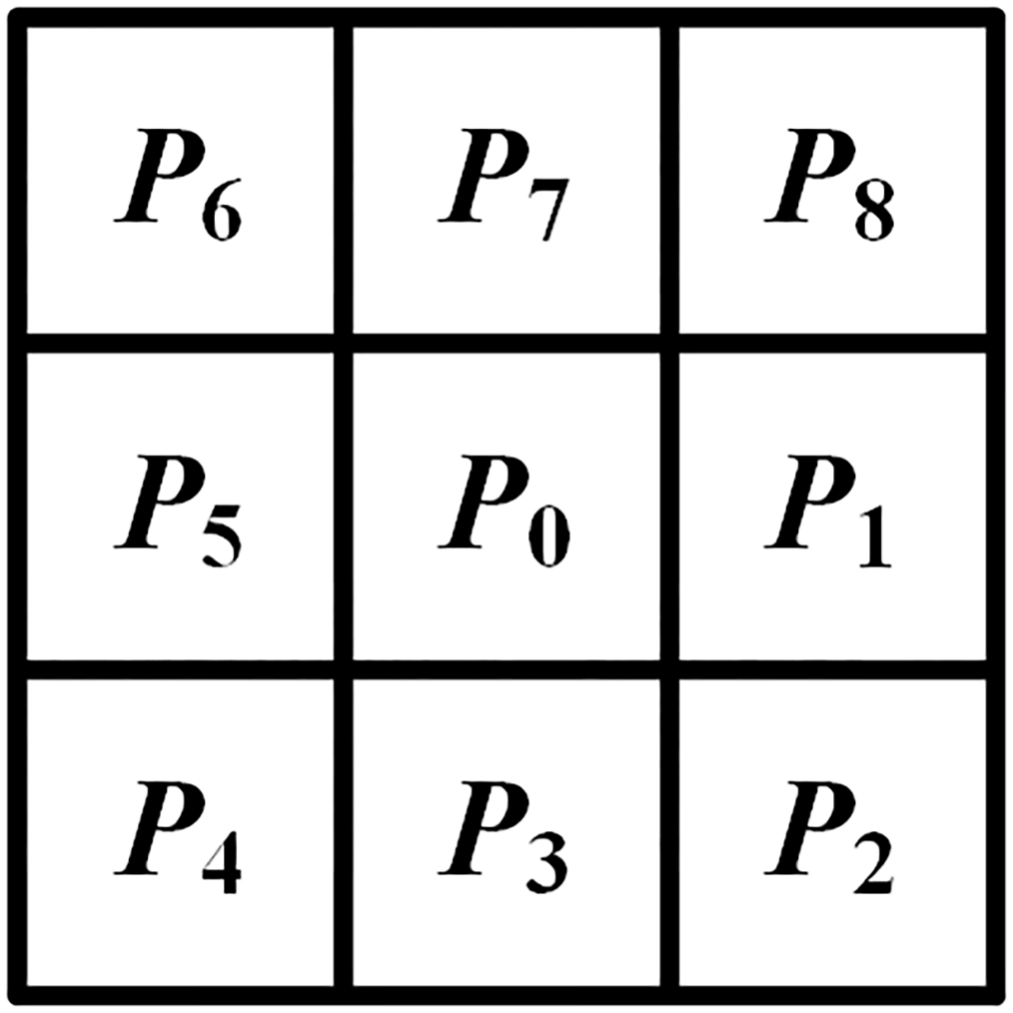
3×3 exploration grid.


The algorithm for skeletonization based on thinning operates on binary images where pixels labeled ‘1’ denote the target pixels, and ‘0’ designates the background pixels. In this binary context, a pixel manifesting a value of ‘1’ is delineated as a boundary pixel of the object if it is adjacent to at least one ‘0’ value pixel within its octal neighborhood. The iterative process begins at these boundary pixels, methodically stripping away pixels from the perimeter of the object that conform to predefined conditions. In the initial phase, the skeletonization algorithm designates a boundary pixel, denoted as *P*_0_, to act as the central pixel. This pixel is encircled by eight neighboring pixels, labeled *P*_1_ to *P*_8_, which are arranged clockwise to constitute a 3×3 exploration grid. The numbering of this 8-connected neighborhood is shown in **Figure 7**. Following this setup, the algorithm evaluates whether *P*_0_ fulfills certain predefined criteria as detailed in Equation 1. Upon satisfying these criteria, *P*_0_ is flagged for exclusion in the subsequent iteration of skeleton pruning.

(1) $$\begin{array}{c} 2\leq \text{N}\left({\text{P}}_{0}\right)\leq 6\\ \text{S}\left({\text{P}}_{0}\right)=1\\ {\text{P}}_{7}\times {\text{P}}_{1}\times {\text{P}}_{3}=0\\ {\text{P}}_{1}\times {\text{P}}_{3}\times {\text{P}}_{5}=0 \end{array}$$

Here, *N*(*P*_0_) denotes the number of pixels with a value of 1 within the 8-neighborhood of *P*_0_, and *S*(*P*_0_) represents the number of transitions from 0 to 1 among the eight neighboring pixels around *P*_0_ when they are considered in a clockwise direction.

The decision criteria are modified such that the product of *P*_2_, *P*_4_, and *P*_8_ equals zero as well as the product of *P*_1_, *P*_2_, and *P*_3_ equals zero. Following the establishment of these conditions, a subsequent assessment is undertaken to identify and subsequently prune pixels conforming to these established decision metrics. The precise conditions governing these evaluations are delineated in Equation 2.


(2) $$\begin{array}{c} 2\leq \text{N}\left({\text{P}}_{0}\right)\leq 6\\ \text{S}\left({\text{P}}_{0}\right)=1\\ {\text{P}}_{7}\times {\text{P}}_{1}\times {\text{P}}_{5}=0\\ {\text{P}}_{7}\times {\text{P}}_{3}\times {\text{P}}_{5}=0 \end{array}$$

After conducting two successive rounds of condition evaluation, one iteration of the algorithm concludes. This sequence is reiterated persistently until a state is reached where none of the pixel points fulfill the criteria for assessment. This iterative process culminates in the derivation of the skeleton of the target object.

#### 2.4.4 Image processing workflow

**Figure 8:**
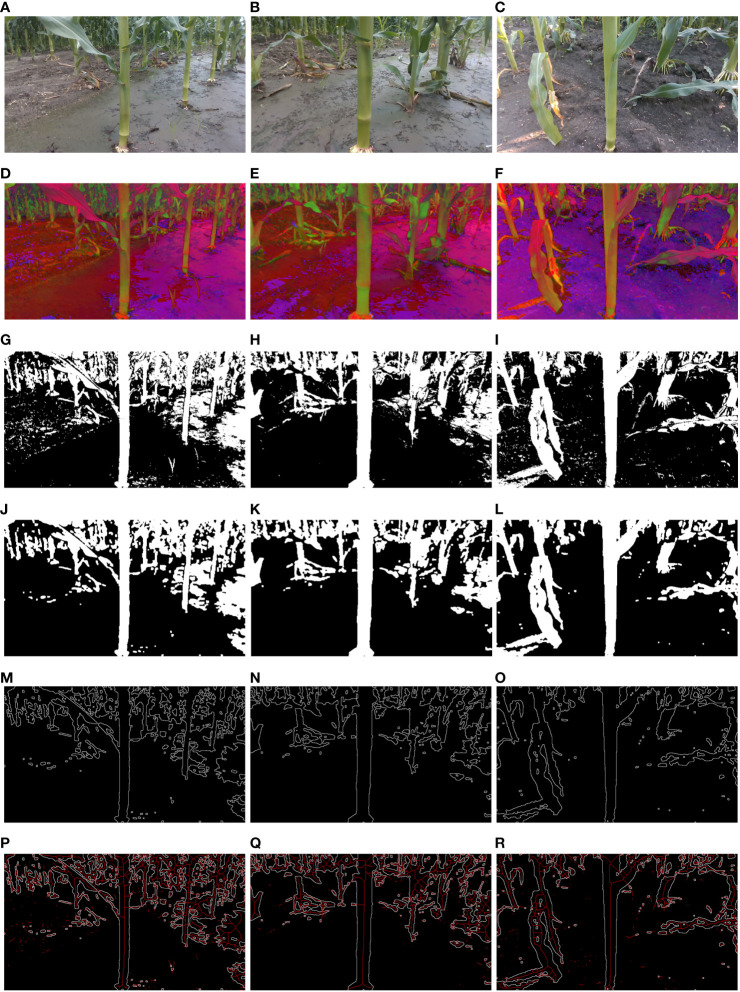
Image processing process: **(A–C)** Field maize images; **(D–F)** HSV images; **(G–I)** HSV+Otsu images; **(J–L)** Denoised images obtained through median filtering, binarization, and morphological opening operations; **(M–O)** Maize stem contour images obtained via internal gradient algorithms; **(P–R)** Skeleton images obtained through skeleton extraction algorithms.

Three distinct image sets were randomly sampled from a collection of sixty field maize image groups for experimental analysis. The field maize images, images based on the HSV color space, images processed with the HSV and Otsu algorithms, images of maize stem processed using denoising algorithms, internal gradient algorithms, and skeleton extraction algorithms are presented in **Figure 8**.

#### 2.4.5 Coordinate extraction and stem diameter measurement

**Figure 9:**
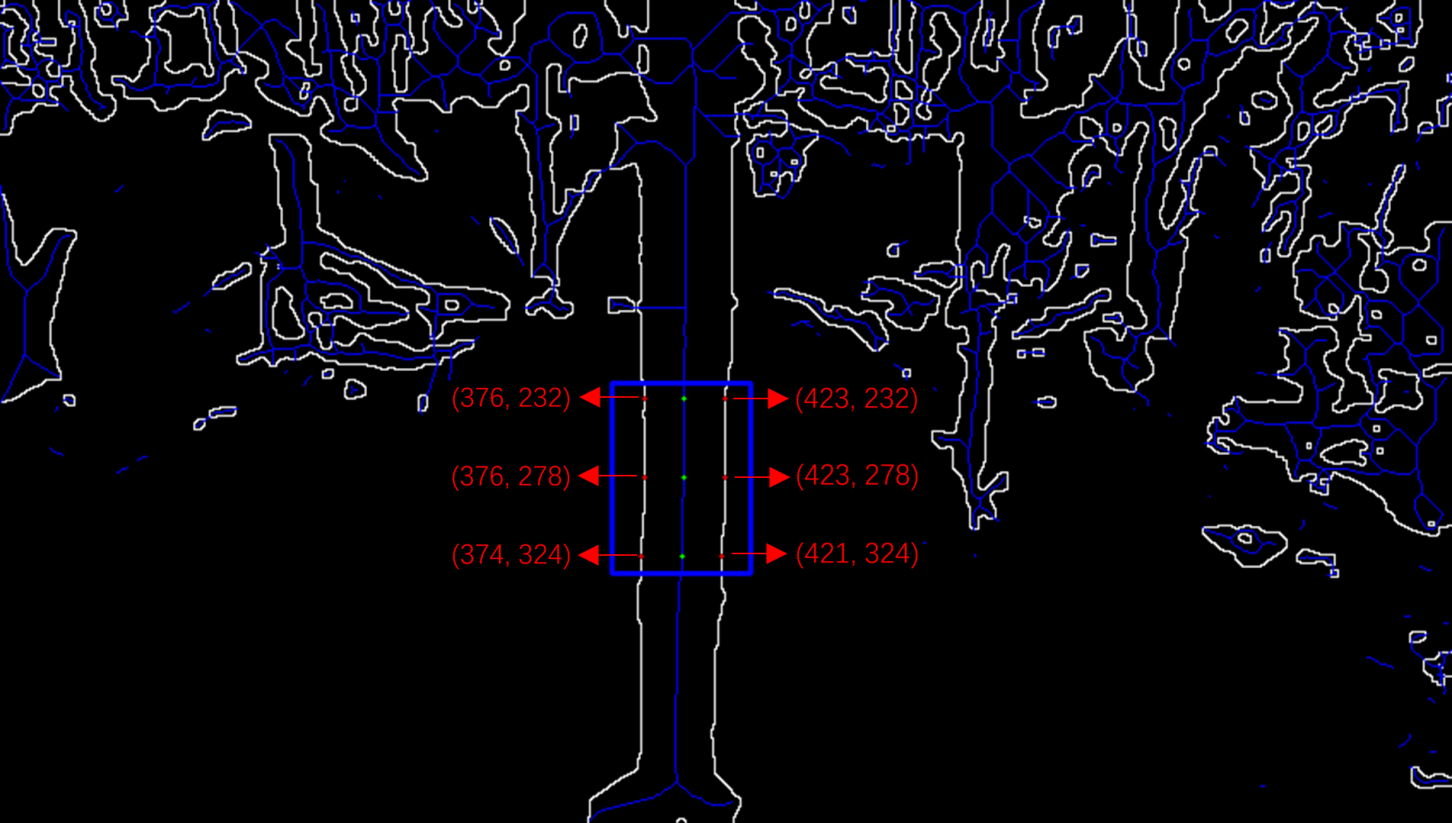
2D coordinate extraction process.

In the maize stem skeleton images, considering that the diameter of the second internode can directly affect the maize’s lodging resistance, the second internode of the maize stem has been designated as the area of interest (Zhang et al., 2018). In the specified region of interest, coordinate extraction in two dimensions is assisted by utilizing the cv2.inRange function from the OpenCV library within a Python environment. The process is fully automated to obviate manual intervention. Initially, a point located on the skeletal line within the defined region of interest is identified and annotated on the image. Subsequently, a horizontal line emanating from this reference point is extended to ascertain the intersection with the contour of maize stem. Concluding this step, the points of intersection are labeled on the image, and the 2D pixel coordinates corresponding to these intersections are meticulously documented. In the region of interest, the extraction procedure is performed three times to confirm the accuracy and consistency of the stem diameter measurements. The process of 2D coordinate extraction is illustrated in **Figure 9**.

To transform 2D pixel coordinates into their 3D world coordinates, a synthesis of depth data, intrinsic camera parameters, and the 2D pixel coordinates is essential to achieve the back-projection transformation from pixel to world space. After the back-projection transformation, 3D world coordinates based on the coordinate system of the color flow camera can be obtained. The transformation formula for back-projection is delineated in Equation 3. The intrinsic parameters characterizing the Intel RealSense D435i camera with a resolution of 848×480 are itemized in **Supplementary Table 1**.


(3) $$\begin{array}{c} \text{Z}=\text{d}\\ \text{X}=\frac{\text{x}-{\text{c}}_{\text{x}}}{{\text{f}}_{\text{x}}}\times \text{Z}\\ \text{Y}=\frac{\text{y}-{\text{c}}_{\text{y}}}{{\text{f}}_{\text{y}}}\times \text{Z} \end{array}$$

Here, (*x*,*y*) represent pixel coordinates on the 2D image plane, d corresponds to depth information for those pixel points in 3D space, (*c_x_*,*c_y_*) correspond to principal point coordinates within camera intrinsic parameters, and *f_x_* and *f_y_* denote camera focal lengths along *x* and *y* axes, respectively.

**Figure 10:**
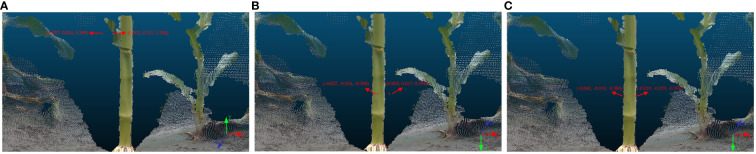
3D world coordinates in different coordinate systems: **(A)** 3D world coordinates in the right-handed coordinate system; **(B)** 3D world coordinates in the color stream camera coordinate system; **(C)** 3D world coordinates in the depth stream camera coordinate system.


Furthermore, for precise mapping of spatial positions of 3D world coordinates onto the maize stem point cloud, transformation of the 3D world coordinates from the right-handed coordinate system, utilized by OpenCV, to the coordinate system of the color stream camera of the Intel RealSense Viewer is imperative. This necessitates an inversion operation. Thereafter, a rigid transformation is performed to transfer the 3D world coordinates from the color stream camera coordinate system to the depth stream camera coordinate system. Initially, OpenCV employs a right-handed coordinate system by convention, which diverges from the color stream camera coordinate system defined by the Intel RealSense Viewer. Consequently, this research necessitates inverting the y and z-axis values of the 3D world coordinates to conform to the coordinate system defined for the color stream camera. In addition, the Intel RealSense Viewer generates the 3D point cloud of the maize stem using the coordinate system of the depth stream camera, distinct from the coordinate system for the color stream camera that locates the 3D world coordinates. To accurately map 3D world coordinates within the point cloud, this study applies rigid transformation techniques to convert the 3D world coordinates from the color stream camera coordinate system to that of the depth stream camera. Upon transformation, the 3D world coordinates are delineated in red within the point cloud, corroborating the precision of spatial position representation of the method employed to acquire field maize stem diameter using depth data. The algorithm governing rigid transformation is encapsulated in Equation 4, camera extrinsic parameters are enumerated in **Supplementary Table 2**, and **Figure 10** illustrates a comparative schematic of the conversion between coordinate systems.

(4) $${\text{p}}^{,}=\text{Rp}+\text{t}$$

Here, *R* represents camera rotation matrix, *t* denotes camera translation vector, *p* is the 3D world coordinate in color stream camera coordinate system, and *p’* is the 3D world coordinate in depth stream camera coordinate system.

**Figure 10** illustrates the process of obtaining stem diameter measurements through the computation of Euclidean distance between pairs of 3D world coordinates. The formula for the Euclidean distance between two points in 3D space is presented as Equation 5.


(5) $$\text{d}=\sqrt{{\left({\text{x}}_{2}-{\text{x}}_{1}\right)}^{2}+{\left({\text{y}}_{2}-{\text{y}}_{1}\right)}^{2}+{\left({\text{z}}_{2}-{\text{z}}_{1}\right)}^{2}}$$

Here, (*x*_1_,*y*_1_,*z*_1_) and (*x*_2_,*y*_2_,*z*_2_) represent the 3D world coordinates of the two points, respectively, with d denoting the distance between them.

In conclusion, the depth data procured from the RGB-D camera has been effectively employed to determine the diameter of maize stems in situ. This method furnishes significant data support for further investigative endeavors.

### 2.5 Evaluation metrics

To ascertain the accuracy of the method for deriving maize stem diameter measurements *in situ* from depth information, this study executed manual measurements of maize stem diameters using a Vernier caliper and conducted a comparative analysis between these manual measurements and the measurements derived from depth information. The Mean Absolute Percentage Error *(MAPE)*, Mean Absolute Error *(MAE)*, Root Mean Square Error *(RMSE)*, and the coefficient of determination *(R*²) serve as metrics to evaluate accuracy. The computational formulas for these indices are delineated in Equations 6–9.


(6) $$\text{MAPE}=\frac{1}{\text{n}}\sum_{\text{i}=1}^{\text{n}}\frac{\left|{\text{w}}_{\text{i}}-{\text{k}}_{\text{i}}\right|}{{\text{k}}_{\text{i}}}\times 100\%$$

(7) $$\text{MAE}=\frac{1}{\text{n}}\sum_{\text{i}=1}^{\text{n}}\left|{\text{w}}_{\text{i}}-{\text{k}}_{\text{i}}\right|$$

(8) $$\text{RMSE}=\sqrt{\frac{1}{\text{n}}\sum_{\text{i}=1}^{\text{n}}{\left({\text{w}}_{\text{i}}-{\text{k}}_{\text{i}}\right)}^{2}}$$

(9) $${\text{R}}^{2}=1-\frac{\sum_{\text{i}=1}^{\text{n}}{\left({\text{k}}_{\text{i}}-{\text{w}}_{\text{i}}\right)}^{2}}{\sum_{\text{i}=1}^{\text{n}}{\left({\text{k}}_{\text{i}}-\bar{\text{k}}\right)}^{2}}$$

Here, *n* represents the number of plant samples, *w_i_* represents the stem diameter measurements based on depth information, *k_i_* denotes the manually measured values, and $\bar{k}$ is the average value of the manual measurements of maize stem diameter.

## 3 Results

### 3.1 Error analysis of maize stem diameter measurements based on depth information

A random selection of 60 sets of maize plants was utilized as the experimental material. The maize stem diameters for these sets were obtained based on depth information. The error analysis data comparing stem diameter measurements with manual measurements are shown in **Table 1**.

**Table 1:** Comparison between stem diameter measurements obtained from depth information and manual measurements.

Number	Measured Stem Diameter/mm	True Stem Diameter/mm	Absolute Error/mm	Number	Measured Stem Diameter/mm	True Stem Diameter/mm	Absolute Error/mm
1	34.72	37.42	2.70	31	22.72	21.66	1.06
2	26.48	27.06	0.58	32	25.64	25.55	0.09
3	31.35	32.00	0.65	33	20.93	20.69	0.24
4	39.10	40.08	0.98	34	21.72	22.29	0.57
5	35.76	36.07	0.31	35	24.12	23.89	0.23
6	22.79	26.47	3.68	36	22.89	21.92	0.97
7	34.97	36.01	1.04	37	21.49	23.53	2.04
8	30.12	30.91	0.79	38	26.74	26.34	0.40
9	29.12	30.10	0.98	39	20.27	20.11	0.16
10	23.80	23.71	0.09	40	26.82	26.62	0.20
11	21.88	21.92	0.04	41	27.66	28.15	0.49
12	32.24	31.48	0.76	42	21.69	21.54	0.15
13	27.60	27.52	0.08	43	23.01	22.46	0.55
14	25.16	25.90	0.74	44	20.25	20.49	0.24
15	30.26	29.72	0.54	45	21.39	22.37	0.98
16	30.79	30.43	0.36	46	23.37	23.27	0.10
17	28.21	30.76	2.55	47	21.34	21.58	0.24
18	39.76	40.61	0.85	48	26.50	24.36	2.14
19	28.50	29.61	1.11	49	20.07	17.55	2.52
20	21.48	21.67	0.19	50	20.05	19.38	0.67
21	27.86	28.86	1.00	51	17.17	17.89	0.72
22	22.69	23.56	0.87	52	21.43	21.52	0.09
23	22.41	23.47	1.06	53	24.82	23.35	1.47
24	24.71	24.76	0.05	54	24.58	22.64	1.94
25	22.04	21.85	0.19	55	25.87	25.62	0.25
26	24.91	24.51	0.40	56	22.81	22.60	0.21
27	25.66	26.47	0.81	57	21.07	20.67	0.40
28	23.68	23.77	0.09	58	21.87	21.74	0.13
29	22.42	21.24	1.18	59	18.46	17.49	0.97
30	21.62	21.55	0.07	60	19.91	19.60	0.31

Analysis of data from **Table 1** reveals that the *MAPE* for the sampled set of 60 maize stem diameters is 3.01%, the *MAE* measures at 0.75mm, and the *RMSE* stands at 1.07mm. Given that the *MAE* is below 1mm and the *MAPE* does not exceed 3.1%, measurements of maize stem diameters based on depth information are shown to be accurate.

### 3.2 Comparative error analysis of maize stem diameter measurements based on the pinhole imaging principle and the method described in this paper

**Figure 11:**
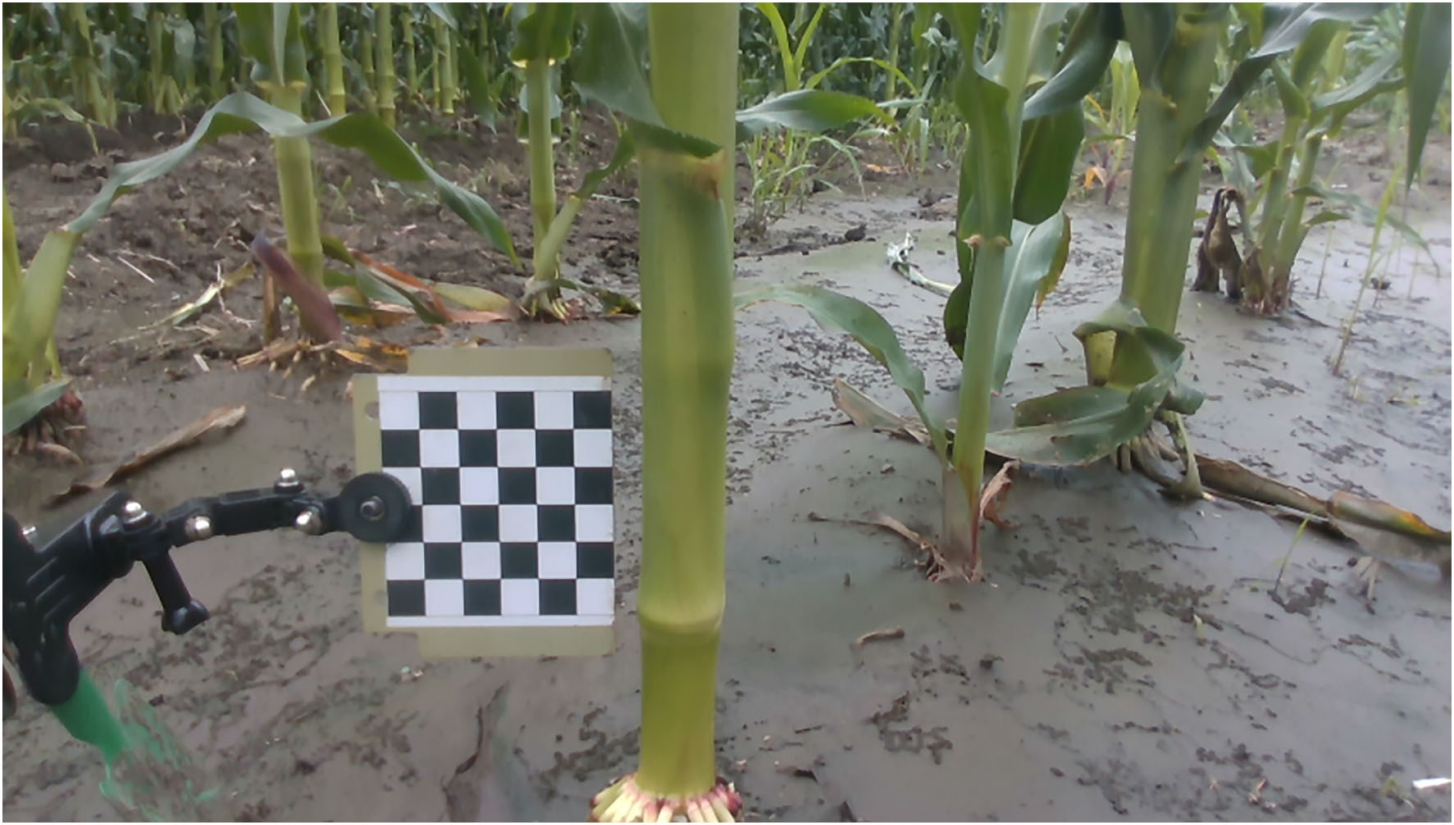
Field maize images obtained based on the pinhole imaging principle.

Previous research has effectively measured the diameter of maize stems in the field utilizing a checkerboard for reference, applying the pinhole imaging principle (Zhou et al., 2023a). The present study seeks to evaluate the efficacy of measuring maize stem diameters in the field by comparing the pinhole imaging principle with the method proposed herein. Specifically, when the camera captured images of field maize using the method outlined in this paper, images of field maize with a checkerboard were also taken at the same location and angle. These two measurement tasks were completed consecutively within the same day to ensure consistency in experimental conditions. To augment the precision of measurements derived from the pinhole imaging principle, this study introduced enhancements to the experimental apparatus. Specifically, the checkerboard was fixed using a triaxial adjustment arm, which aids in precisely regulating its tilt angle to ensure that the checkerboard is as parallel as possible to the imaging plane of the camera. Images of field maize obtained using the pinhole imaging principle are shown in **Figure 11**. A total of 60 maize plant samples were selected as experimental material, which are the same sets as those used in Section 3.1. The diameters of maize stems from these samples were quantified employing the pinhole imaging principle. The error analysis data comparing stem diameter measurements with manual measurements are presented in **Table 2**.

**Table 2:** Comparison of stem diameter measurements obtained using the pinhole imaging principle and manual measurements.

Number	Measured Stem Diameter/mm	True Stem Diameter/mm	Absolute Error/mm	Number	Measured Stem Diameter/mm	True Stem Diameter/mm	Absolute Error/mm
1	34.57	37.42	2.85	31	23.06	21.66	1.40
2	26.05	27.06	1.01	32	26.56	25.55	1.01
3	29.71	32.00	2.29	33	21.94	20.69	1.25
4	41.17	40.08	1.09	34	22.00	22.29	0.29
5	36.92	36.07	0.85	35	26.43	23.89	2.54
6	22.04	26.47	4.43	36	24.47	21.92	2.55
7	32.50	36.01	3.51	37	20.83	23.53	2.70
8	32.05	30.91	1.14	38	28.16	26.34	1.82
9	28.54	30.10	1.56	39	20.28	20.11	0.17
10	24.55	23.71	0.84	40	28.40	26.62	1.78
11	22.80	21.92	0.88	41	30.28	28.15	2.13
12	32.93	31.48	1.45	42	23.25	21.54	1.71
13	30.91	27.52	3.39	43	23.80	22.46	1.34
14	24.47	25.90	1.43	44	19.13	20.49	1.36
15	31.43	29.72	1.71	45	24.75	22.37	2.38
16	29.78	30.43	0.65	46	21.55	23.27	1.72
17	26.39	30.76	4.37	47	25.00	21.58	3.42
18	43.04	40.61	2.43	48	28.75	24.36	4.39
19	25.83	29.61	3.78	49	21.30	17.55	3.75
20	21.19	21.67	0.48	50	20.21	19.38	0.83
21	31.30	28.86	2.44	51	17.27	17.89	0.62
22	23.62	23.56	0.06	52	20.81	21.52	0.71
23	22.22	23.47	1.25	53	25.75	23.35	2.40
24	23.85	24.76	0.91	54	29.29	22.64	6.65
25	20.60	21.85	1.25	55	26.59	25.62	0.97
26	24.64	24.51	0.13	56	23.10	22.60	0.50
27	24.47	26.47	2.00	57	20.00	20.67	0.67
28	22.89	23.77	0.88	58	23.68	21.74	1.94
29	22.95	21.24	1.71	59	19.47	17.49	1.98
30	22.50	21.55	0.95	60	22.25	19.60	2.65

According to the data in **Table 2**, the *MAPE*, *MAE*, and *RMSE* for the 60 sets of maize stem diameter measurements are 7.34%, 1.82mm, and 2.22mm, respectively. A comparative analysis with the errors obtained from the maize stem diameter measurements using the method described in this paper reveals that the values of *MAE*, *MAPE*, and *RMSE* derived from depth information exhibit lower figures. Specifically, the *MAPE*, *MAE*, and *RMSE* demonstrated reductions of 4.33%, 1.07mm, and 1.15mm, respectively. Given the aforementioned analysis, it is concluded that the precision of field maize stem diameter measurements derived from depth information surpasses that obtained by methods based on the pinhole imaging principle.

**Figure 12:**
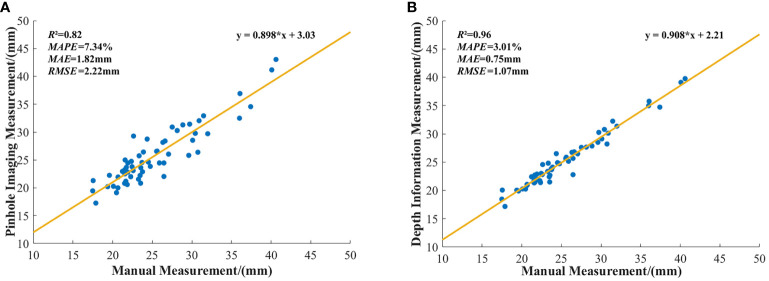
Linear fitting between stem diameter measurements and manual measurements: **(A)** Linear fitting between stem diameter measurements obtained using the pinhole imaging principle and manual measurements. **(B)** Linear fitting between stem diameter measurements obtained from depth information and manual measurements.

To visually illustrate the differences between stem diameter measurements obtained through the pinhole imaging principle and manual measurements, as well as to delineate the variance between measurements derived from the method described in this paper and those obtained manually, this study performed a linear fitting of these datasets. The outcomes of this fitting are depicted in **Figure 12**.

The linear fit results shown in **Figure 12** indicate that the *R*² for measurements based on the pinhole imaging principle is 0.82, whereas the *R*² for measurements based on depth information is 0.96. These findings substantiate the superior precision of the depth information-based method for determining field maize stem diameters over those obtained by the pinhole imaging principle.

**Figure 13:**
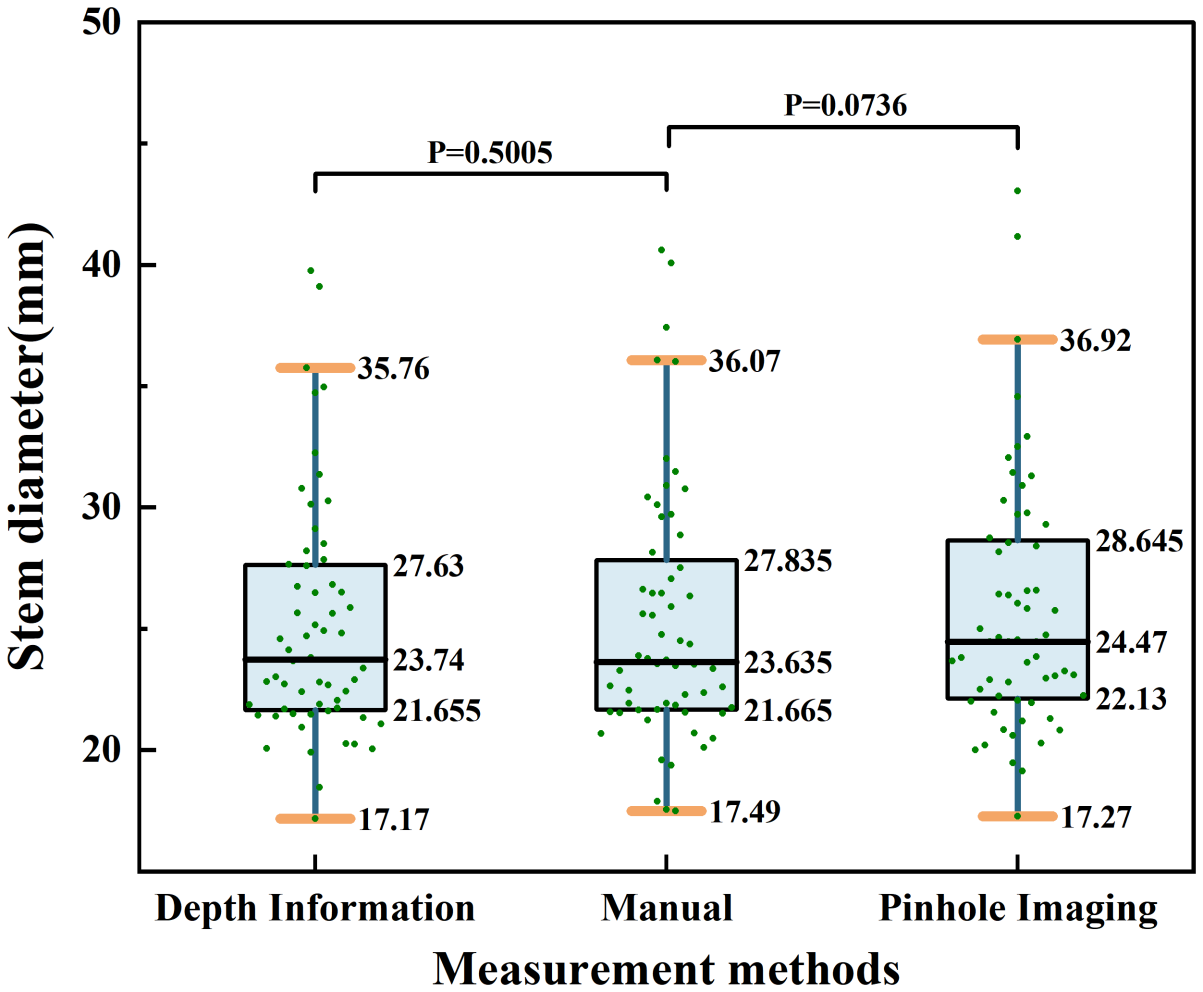
Distribution of stem diameter measurement results based on depth information, manual measurement, and the pinhole imaging principle.

Furthermore, to more comprehensively compare the differences between the two measurement methods in terms of precision, stability, and consistency, this study utilized a combination of box plots and scatter plots to display the distribution of stem diameter measurements based on depth information, manual measurement, and the principle of pinhole imaging. On this basis, statistical difference analysis was conducted. Since all measurement results did not conform to a normal distribution, non-parametric Wilcoxon signed-rank tests were used for the difference analysis. The distribution of results is shown in **Figure 13**. As shown in **Figure 13**, the comparison between stem diameter values obtained from depth information and those obtained through manual measurement results in a P-value of 0.5005; the comparison between stem diameter values obtained from the principle of pinhole imaging and those obtained through manual measurement results in a P-value of 0.0736. These results do not provide sufficient statistical evidence to suggest a significant difference between the methods of measurement based on depth information or the principle of pinhole imaging and the method of manual measurement.

To further compare the consistency between the two measurement methods, this study employed Lin’s Concordance Correlation Coefficient (CCC) to analyze the two methods. This coefficient, by comprehensively evaluating the covariance of the measurements and the differences between their respective means, can effectively reflect the consistency between the results of the two measurement methods. The closer the value of CCC is to 1, the better the consistency between the two measurement methods. The calculation formula for the CCC is delineated in Equation 10.


(10) $${\text{ρ}}_{\text{c}}=\frac{2\text{ρ}{\text{σ}}_{\text{x}}{\text{σ}}_{\text{y}}}{\sigma_{\text{x}}^{2}+\sigma_{\text{y}}^{2}+{\left({\text{μ}}_{\text{x}}-{\text{μ}}_{\text{y}}\right)}^{2}}$$

Here, *ρ* represents the Pearson correlation coefficient between the two sets of measurements, *σ_x_* and *σ_y_* are the standard deviations of the two sets of measurements, *μ_x_* and *μ_y_* are the means of the two sets of measurements, *ρ_c_* is Lin’s Concordance Correlation Coefficient.

Through calculation, it can be determined that the CCC between stem diameter values obtained from depth information and manual measurements is 0.978, while the CCC between stem diameter values obtained from the principle of pinhole imaging and manual measurements is 0.909. These results indicate that, compared to the principle of pinhole imaging, the measurement method based on depth information shows a closer alignment with manual measurement results, demonstrating better consistency.

Furthermore, as indicated by the box plots in **Figure 13**, the distribution widths for maize stem diameters measured using depth information, manual techniques, and the pinhole imaging principle are 18.59mm, 18.58mm, and 19.65mm, respectively. The interquartile ranges are 5.78mm, 6.17mm, and 6.52mm, respectively, and the medians are 23.74mm, 23.64mm, and 24.47mm, respectively. In comparison to the pinhole imaging principle, the median values of maize stem diameters measured using depth information more closely align with those obtained by manual measurement, further validating its advantage in precision. Additionally, the distribution width and interquartile range of maize stem diameters gathered from depth information are also closer to those from manual measurements, indicating its superior performance in terms of stability and consistency. In summary, from the perspectives of accuracy, stability, and consistency, the method of acquiring field maize stem diameters based on depth information has demonstrated superior performance.

## 4 Discussion

In response to the constraints presented by conventional, laborious phenotypic measurements in agronomic research, this study proposes an innovative method for the quantification of maize stem diameter *in situ* employing depth information from an RGB-D camera. RGB images, depth maps, and 3D point clouds of maize stems in the field were captured using an Intel RealSense D435i camera. An effective solution for the precise alignment of RGB and depth images is provided by the rs.align class within the pyrealsense2 library. Furthermore, the automation of acquiring 2D pixel coordinates is enhanced by utilizing a skeleton extraction algorithm based on thinning techniques. The integration of depth information with intrinsic parameters of the camera enables the transformation of 2D pixel coordinates into 3D world coordinates through a back-projection transformation. Subsequently, through rigid transformation techniques, these 3D world coordinates are precisely mapped onto the 3D point cloud. In conclusion, the quantification of maize stem diameter was accomplished by computing the Euclidean distance between pairs of 3D world coordinates. The empirical outcomes substantiated the precision, reliability, and uniformity of the proposed method for acquiring field maize stem diameters utilizing depth information derived from an RGB-D camera.

Relative to analogous technologies, the technique for measuring the diameter of maize stems in the field via depth information from an RGB-D camera has exhibited specific advantages. Initially, lidar technology has been demonstrated to be effective for acquiring the diameter of maize stems. Miao et al. (2022) collected 3D point cloud data of maize across extensive fields employing terrestrial laser scanning and obtained the stem diameters by applying elliptical fitting techniques, achieving an *R*² in excess of 0.8. Ma et al. (2019) employed a handheld lidar to obtain the diameter of potted maize stems, achieving an *R*² of 0.89. Nonetheless, given that lidar technology utilizes laser beams to gauge object surface distances, its utility is primarily confined to the acquisition of 3D point cloud data of maize plants, with an inherent limitation in gathering chromatic information. Moreover, the production and maintenance costs associated with this technology are considerable. In contrast, the Intel RealSense D435i camera employed in this research possesses the capability to concurrently capture color imagery, depth maps, and 3D point clouds of maize stems. Color data play a pivotal role in aiding researchers to diagnose crop diseases and infestations (Deng et al., 2020). Additionally, this camera is not only economical and portable but also amenable to further development. Additionally, cameras based on the TOF principle can also be employed to measure the diameter of maize stems. Chaivivatrakul et al. (2014) utilized a TOF camera to collect 3D point cloud data of indoor potted maize and successfully extracted the stem diameter using elliptical fitting techniques, with an *R*² of 0.84. Bao et al. (2019) captured 3D point cloud data for maize in field conditions utilizing a side-view TOF camera and extracted the stem diameter through a method based on 3D skeletal lines. However, the *R*² was a mere 0.27, indicating lower precision. The observed discrepancy in accuracy between the two referenced studies may be ascribed to the inherent resolution constraints of the TOF camera coupled with its pronounced susceptibility to ambient natural light, culminating in suboptimal measurements within outdoor settings (Kazmi et al., 2014). Compared to the time-of-flight imaging technology of TOF cameras, the Intel RealSense D435i camera employs stereo vision technology, which enables it to provide more robust high-resolution depth data in outdoor environments (Vit and Shani, 2018). Conclusively, contact measurement techniques are also viable for determining the diameter of maize stems. Atefi et al. (2020) utilized a robotic system fitted with fixtures to measure the diameters of maize and sorghum stems under controlled laboratory conditions, yielding *R*² values of 0.98 and 0.99, respectively. Such precision underscores the high accuracy of the measurement methods. Nevertheless, contact measurement methods require a high level of operator skill, and any mishandling might inflict damage on the maize stems. By contrast, this study utilizes non-invasive imaging technologies for the measurement of maize stem diameters, a method that obviates the need for physical contact with the stems and consequently mitigates the risk of damage to the crops.

In the complex field environment, developing an imaging system that can adapt to diverse environmental factors has always been a scientific challenge. Although RGB-D cameras based on depth information have successfully acquired the diameter of field maize stems to a certain extent, they also present some issues that require further investigation. The principal challenge encountered in field phenotyping is the substantial effect of ambient illumination on image quality. The Intel RealSense D435i camera, amongst a range of RGB-D imaging devices, manifests reduced sensitivity to light variation. Nevertheless, its operational performance can be compromised under the intense illumination characteristic of peak midday sun (Vit and Shani, 2018). Future research will employ near-infrared filters to optimize camera performance in bright light conditions (Gai et al., 2015; He et al., 2021). Additionally, the current data collection is limited to clear weather conditions and does not encompass overcast conditions. Therefore, future research will consider data collection under various weather conditions to more comprehensively evaluate the applicability and robustness of the method presented in this paper. In addition, this study was conducted using a relatively sparse planting pattern, which, to some extent, reduced the interference from adjacent plants on the experiments. However, it also lacked observation of plants under conventional planting patterns. Therefore, future research will continue to optimize the experimental design, thereby exploring the applicability of the methods presented in this paper under conventional planting patterns. Moreover, while side-view imaging technology facilitates the acquisition of the 3D morphology and diameter of maize stems within field conditions, the method of collection from a single angle makes it difficult to present a comprehensive 3D phenotype of the maize stems. Consequently, the pursuit of a method that yields a more holistic 3D phenotype of maize stems will become one of the important directions for future research. Finally, depth information based on RGB-D cameras has proven effective for determining the diameter of maize stems under open field conditions. However, the generalizability of this approach to other crops necessitates additional experimental validation.

## 5 Conclusion

This study proposes a method for acquiring the diameter of maize stems in the field based on depth information from RGB-D cameras. Initially, the contour of the maize stems was obtained through 2D image processing techniques. Subsequently, a skeleton extraction algorithm based on thinning techniques was employed to assist in the acquisition of 2D pixel coordinates. Furthermore, back-projection transformation and rigid transformation techniques are applied to convert 2D pixel coordinates into 3D world coordinates, which are then mapped onto a 3D point cloud. Lastly, the Euclidean distance was applied to calculate the diameter of maize stems, resulting in a *MAPE* of 3.01%, an *MAE* of 0.75mm, a *RMSE* of 1.07mm, and an *R*² of 0.96. Compared with measurement methods based on the pinhole imaging principle, there was a reduction in the *MAE*, *MAPE*, and *RMSE* by 1.07mm, 4.33%, and 1.15mm, respectively. Concurrently, there was an increase of 0.14 in the *R*². The method of acquiring the diameter of field maize stems using depth information from RGB-D cameras maintains the *MAE* within 1.1mm and the *MAPE* within 3.1%, enabling accurate measurement of maize stem diameter. Additionally, this method utilizes non-invasive imaging technology that not only ensures measurement accuracy but also precludes damage to crop surfaces, presenting the possibility to supplant Vernier calipers for monitoring phenotypes of field maize. In the future, should this method be broadly adopted for phenotypic monitoring across diverse crop species, it has the potential to markedly diminish the time and labor required for manual measurements, thereby providing strong technical support for agricultural modernization and precision agriculture.

## Data availability statement

The datasets presented in this study can be found in online repositories. The names of the repository/repositories and accession number(s) can be found below: http://dx.doi.org/10.6084/m9.figshare.25450039.

## Author contributions

JZho: Conceptualization, Funding acquisition, Methodology, Writing – original draft. MC: Data curation, Formal Analysis, Methodology, Writing – original draft. YW: Formal Analysis, Methodology, Visualization, Writing – review & editing. YG: Methodology, Validation, Writing – review & editing. YT: Data curation, Formal Analysis, Writing – review & editing. BJ: Investigation, Writing – review & editing. MW: Project administration, Writing – review & editing. JZha: Supervision, Writing – review & editing. LH: Formal Analysis, Supervision, Writing – original draft.
