# Supplementary material for: Detection of maize stem diameter by using RGB-D cameras’ depth information under selected field condition
Source: Front Plant Sci. 2024 Apr 22;15:1371252. doi: 10.3389/fpls.2024.1371252 (PMC11070473; doi:10.3389/fpls.2024.1371252)
Supplement: Supplementary file 1 [file Table_1.docx]

Supplementary table 1 Intrinsic Parameters of the Intel RealSense D435i at a Resolution of 848×480

| **Parameter Name** | **Symbol** | **Value** |
| --- | --- | --- |
| Horizontal Focal Length | *f*_x_ | 612.882 |
| Vertical Focal Length | *f*_y_ | 611.593 |
| Principal Point x-coordinate | *c*_x_ | 417.970 |
| Principal Point y-coordinate | *c*_y_ | 252.058 |

Supplementary table 2 External parameters for the Intel RealSense D435i

| **Parameter Name** | **Symbol** | **Value** |
| --- | --- | --- |
| Rotation Matrix | *R* | $\left[\begin{matrix} 0.999989 & 0.0016722 & -0.00435564 \\ -0.0016453 & 0.99998 & 0.00617124 \\ 0.00436587 & -0.00616401 & 0.999971 \end{matrix}\right]$ |
| Translation Vector | *t* | [-0.0146885, -0.000213134, 0.000270665] |
